# Removal of Antibiotics From Water with an All-Carbon 3D Nanofiltration Membrane

**DOI:** 10.1186/s11671-018-2555-9

**Published:** 2018-05-10

**Authors:** Guo-hai Yang, Dan-dan Bao, Da-qing Zhang, Cheng Wang, Lu-lu Qu, Hai-tao Li

**Affiliations:** School of Chemistry and Materials Science, Jiangsu Normal University, Xuzhou, 221116 China

**Keywords:** Antibiotics, Graphene oxide, Nanochannels, All-carbon membrane

## Abstract

Recent industrial developments and increased energy demand have resulted in significantly increased levels of environmental pollutants, which have become a serious global problem. Herein, we propose a novel all-carbon nanofiltration (NF) membrane that consists of multi-walled carbon nanotubes (MWCNTs) interposed between graphene oxide (GO) nanosheets to form a three-dimensional (3D) structure. The as-prepared membrane has abundant two-dimensional (2D) nanochannels that can physically sieve antibiotic molecules through electrostatic interaction. As a result, the prepared membrane, with a thickness of 4.26 μm, shows both a high adsorption of 99.23% for tetracycline hydrochloride (TCH) and a high water permeation of 16.12 L m^− 2^ h^− 1^ bar^− 1^. In addition, the cationic dye methylene blue (MB) was also removed to an extent of 83.88%, indicating broad applications of the prepared membrane.

**Electronic supplementary material:**

The online version of this article (10.1186/s11671-018-2555-9) contains supplementary material, which is available to authorized users.

## Background

Recently, pharmaceutical compounds, especially antibiotics, have attracted increasing attention around the world because their occurrence in natural water poses a threat to ecosystems and public health, even at low concentrations [1, 2]. To date, various technologies have been developed with the aim of eliminating antibiotics from the aquatic environment, such as oxidation processes and adsorption [3, 4]. Oxidation processes, such as photocatalysis, sonolysis, and the Fenton reaction, involve complex procedures, whereas membrane-based separations are potentially simpler [5]. However, many currently available membranes for the removal of smaller antibiotic molecules are less effective because they merely operate through a size-exclusion effect [6].

In recent years, carbon-based materials have been used as adsorbents for the removal of antibiotics [7, 8]. Particularly, graphene has also been widely applied to remove pollutants from water due to their one-atom-thick nature, high specific surface areas, and porous structures [9–11]. Graphene oxide (GO) has distinctive structural features [12], excellent hydrophilicity, strong antifouling properties [13], and high mechanical strength. These properties make it suitable for applications in water purification and desalination. In addition, GO may be produced on large scale, in contrast to pristine graphene [14]. However, because of the stacking tendency of GO nanosheets, polymeric materials or large nanoparticles need to be intercalated between them to increase the interlayer spacing [15, 16]. Carbon nanotubes (CNTs), as one-dimensional (1D) materials with excellent properties and compatibilities, have proved to be ideal “nano-wedges” for regulation of the interlayer spacing of GO [17]. Compared with single-walled carbon nanotubes (SWCNTs), multi-walled carbon nanotubes (MWCNTs) provide greater stability under hydrodynamic flow conditions [18]. Moreover, the increased interlayer spacing by intercalation of GO nanosheets with MWCNTs has been proved to enhance water flux. However, undesirable aggregation of CNTs in aqueous solution often hampers application of CNT/GO-based membranes. Conversely, various polyelectrolytes have been used to enhance the dispersion of CNTs through functionalization [19, 20].

In this work, we propose a novel all-carbon nanofiltration (NF) membrane that consists of MWCNTs interposed between GO nanosheets. Poly diallyldimethylammonium chloride (PDDA), as a cationic polyelectrolyte, was grafted onto the MWCNTs to ensure their cationization, imparting strong antifouling properties because of the excellent dispersity. Due to the oxygen-containing functional groups appended both irregularly along the edges and on the surfaces of GO sheets, GO can be regarded as an anionic polyelectrolyte. Thus, the reaction between PDDA-MWCNTs and GO was mainly as a result of charge interaction. The prepared NF membrane was systematically characterized and used as an absorbent for the removal of tetracycline hydrochloride (TCH) and methylene blue (MB) as model organic pollutants. The concentrations of the filtered TCH and MB solutions were determined by UV/Vis spectrophotometry.

## Results and Discussion

**Fig. 1:**
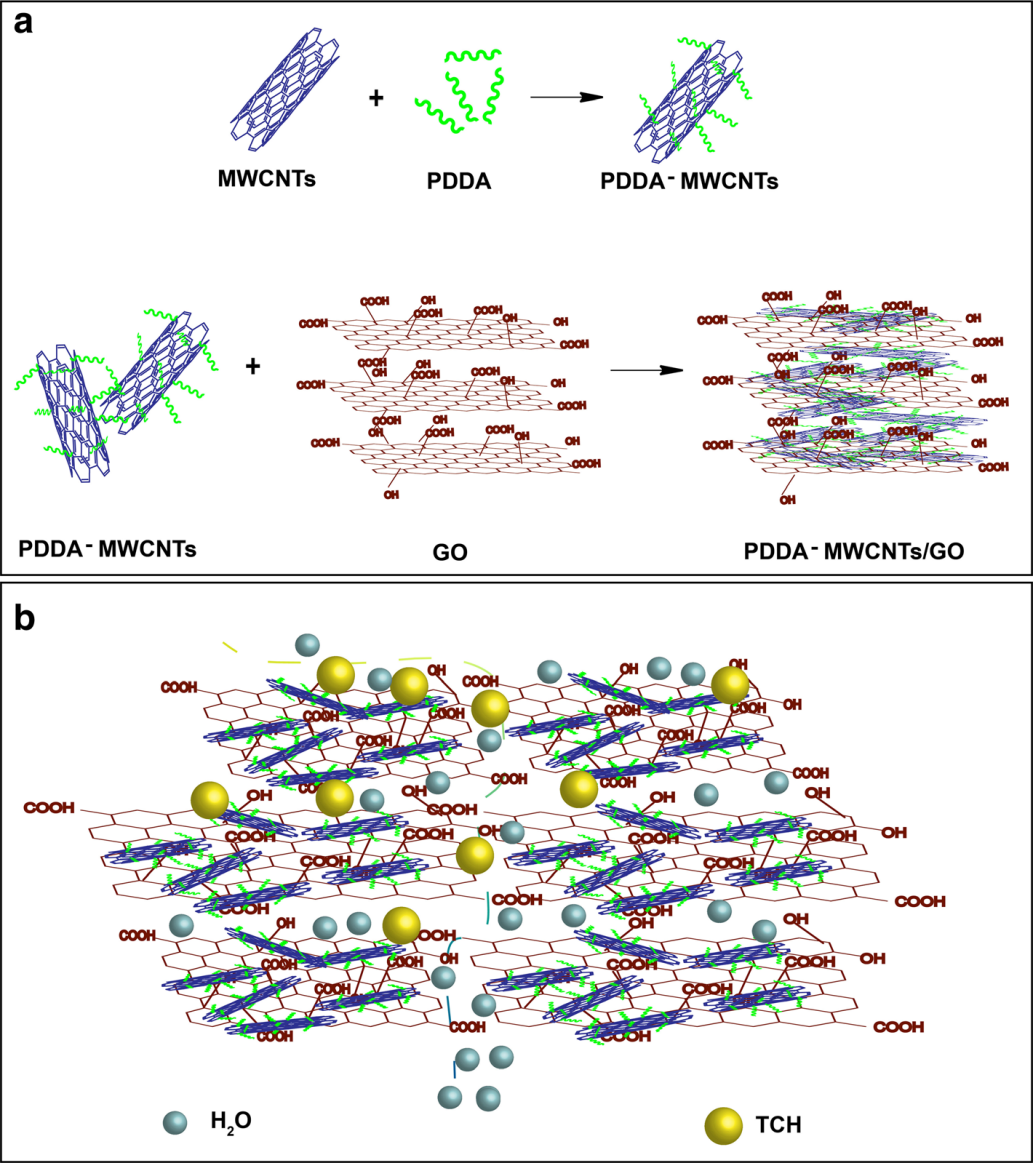
**a** The constructed process for PDDA-MWCNTs/GO membrane. **b** The schematic of adsorption of antibiotics by PDDA-MWCNTs/GO membrane

The MWCNTs/GO hybrid was used to produce a free-standing and flexible membrane based on a simple vacuum filtration method. As illustrated in Fig. 1a, PDDA, the cationic polyelectrolyte, could be easily appended on the surface of the MWCNTs, conferring a positive charge. GO, as an anionic polyelectrolyte, could then react with the positively charged MWCNTs through electrostatic interaction. Finally, an ultrathin membrane was prepared by vacuum filtration of the above dispersion.

The possible adsorption process is illustrated in Fig. 1b. The introduction of MWCNTs between the GO nanosheets opened nanoscale channels, which allowed for improved flow of water molecules [21]. TCH molecules were intercepted in the nanoscale channels due to steric hindrance and their covalent interaction with the functional groups of the prepared all-carbon membrane.

**Fig. 2:**
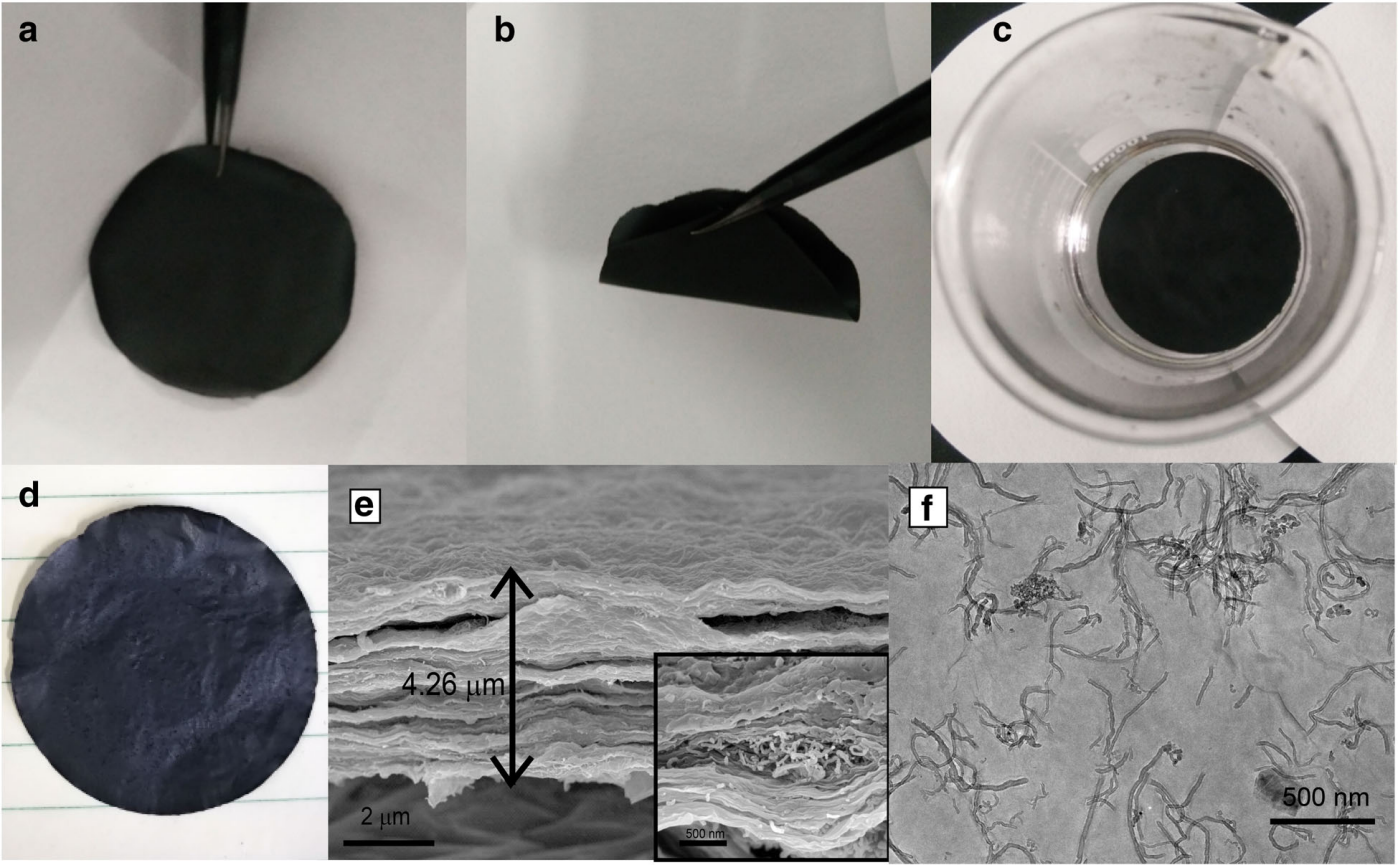
Optical images of PDDA-MWCNTs/GO membrane (**a**), the flexibility of the membrane (**b**), and the stability of membrane in water (**c**). Membrane which had been filtrated for more than 8 cycles (**d**). **e** SEM images of cross-sections of the PDDA-MWCNTs/GO membrane (thickness 4.26 μm). The inset shows a higher magnification image of the PDDA-MWCNTs/GO membrane. **f** TEM images of the ;PDDA-MWCNTs/GO membrane

An optical image of a PDDA-MWCNTs/GO membrane is shown in Fig. 2a. The prepared all-carbon membrane was like cloth and exhibited excellent mechanical flexibility (Fig. 2b). The as-prepared membrane was shown to be hydrophilic by a water contact angle measurement (Additional file 1: Figure S1) [22, 23]. Nevertheless, it proved to be stable when soaked in water (Fig. 2c). Moreover, the membrane could be reused more than seven times without developing any obvious cracks (Fig. 2d).

An SEM image of a cross-section of the prepared membrane is shown in Fig. 2e. The thickness of the membrane was estimated as 4.26 μm, and the MWCNTs were seen to be uniformly inserted between the GO sheets. Moreover, wrinkles on the surface of the prepared membrane were apparent from an AFM image (Additional file 1: Figure S2A), leading to a larger contact area with contaminants. TEM images of the all-carbon membrane revealed that the modified MWCNTs were well dispersed within the GO, in accordance with the SEM results.

**Fig. 3:**
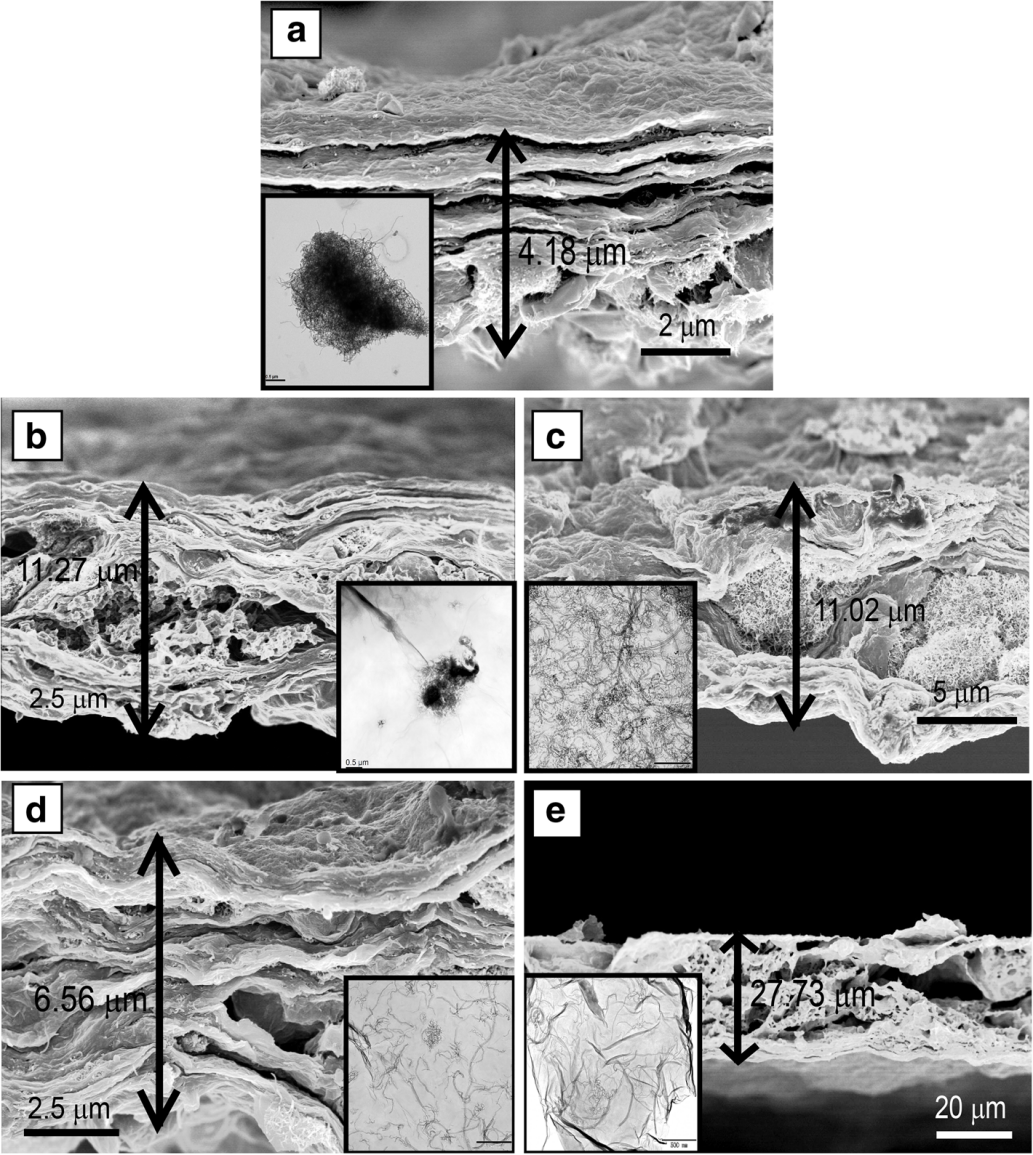
**a** SEM images of cross-sections of the MWCNTs/GO membrane. **b**–**e** SEM images of the cross-sections of the MWCNTs/GO membrane; the inset shows TEM images. The concentration of PDDA is 0, 2, 5, 8, and 20 wt%, respectively

**Fig. 4:**
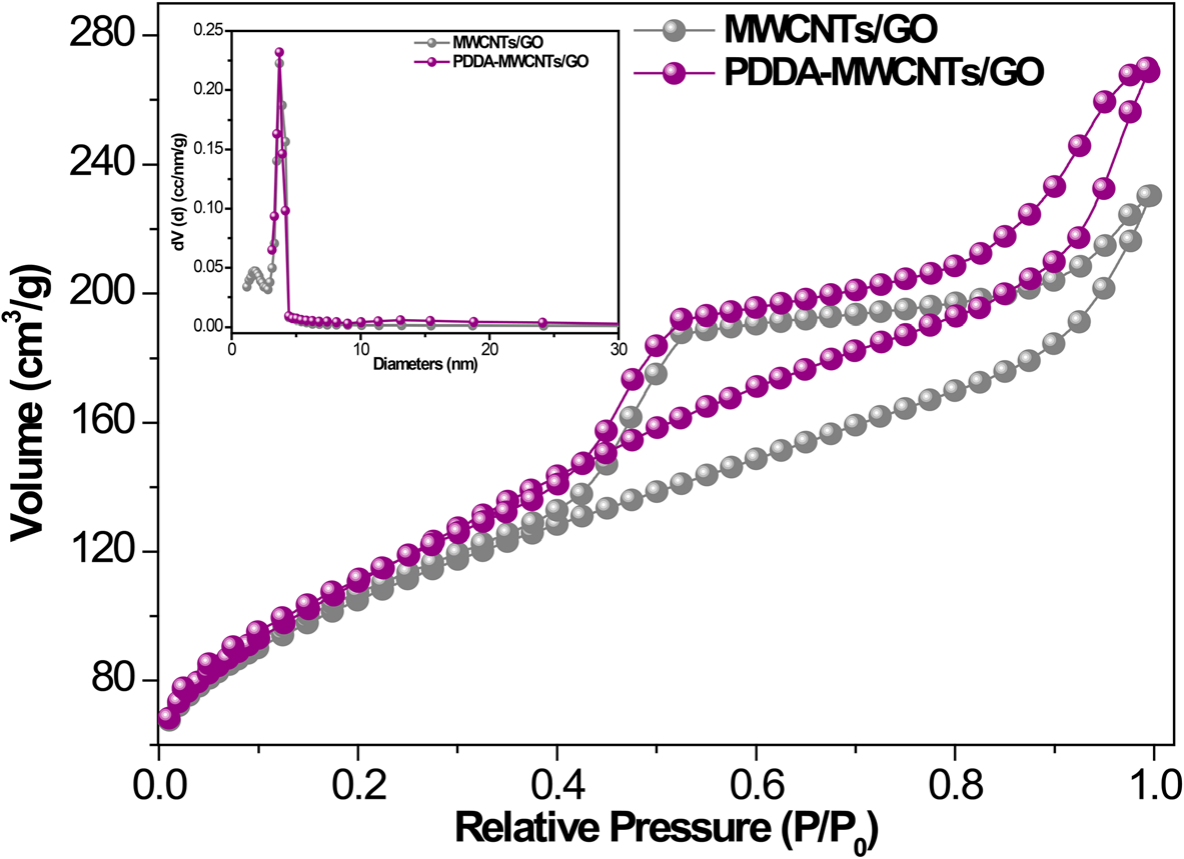
N_2_ adsorption–desorption isotherms of MWCNTs/GO and PDDA-MWCNTs/GO membranes. The inset shows the pore size distribution of the MWCNTs/GO and PDDA-MWCNTs/GO membranes

As shown in Fig. 3a, compared with the GO membrane, the PDDA-MWCNTs/GO membrane exhibited a more porous structure. Variation of the concentration of PDDA influenced the dispersion of MWCNTs in the GO (Fig. 3b–e). Because of the strong adhesive property of PDDA [24], a high concentration thereof (20 wt%) resulted in agglomeration of the MWCNTs (Fig. 3e). However, as can be seen from Fig. 3a–d, PDDA concentrations of 0, 2, 5, or 8 wt% were insufficient to disperse 4 mg of MWCNTs, and the thickness of the membrane was affected. Possible reasons are as follows. Firstly, the membranes were thick partly because MWCNTs were easily agglomerated at low PDDA concentrations. Secondly, non-covalent interaction between a suitable concentration of PDDA and GO sheets can lead to an ultrathin membrane. Thirdly, because MWCNTs become almost enwrapped with long-chain PDDA at excessively high concentrations, large porous structures are formed. However, the mechanism of such pore formation is as yet unknown. It was concluded that a PDDA concentration of 10 wt% gave the optimal dispersion of MWCNTs in GO (Fig. 2e). The morphological characterization including SEM and TEM is presented in Additional file 1: Figure S3. It can be observed that the PDDA is successfully modified into the surface of CNTs, and the thickness of PDDA is around 5.2 nm [25]. Nitrogen adsorption–desorption isotherms were used to characterize the porosities of the MWCNTs/GO and PDDA-MWCNTs/GO membranes (Fig. 4). PDDA-MWCNTs/GO showed increased adsorption–desorption capacity compared with the MWCNTs/GO membrane. The PDDA-MWCNTs/GO NF membrane had a higher specific surface area (402.96 m^2^ g^−1^) than the MWCNTs/GO membrane (378.45 m^2^ g^−1^). Moreover, a typical type IV nitrogen isotherm with hysteresis loops for the prepared NF membrane corroborated its mesoporous properties [26]. The inset image shows the corresponding pore size distribution calculated by the Barrett–Joyner–Halenda (BJH) model, which indicates that the pores of both membranes were about 3–10 nm in diameter, consistent with the N_2_ isotherm.

**Fig. 5:**
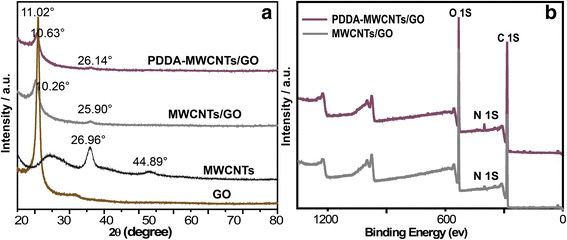
**a** The XRD patterns of GO, MWCNTs, MWCNTs/GO, and PDDA-MWCNTs/GO. **b** XPS spectra of MWCNTs/GO and PDDA-MWCNTs/GO membranes

As shown in Fig. 5a, a characteristic diffraction peak for the GO sample was observed at 11.02° (001), indicating a distance between the nanosheets of 0.80 nm [27], whereas for the MWCNTs peaks were observed at 26.96° (002) and 44.89° [28], in accordance with previous literature reports. After the incorporation of MWCNTs into GO, the characteristic diffraction peaks of both components significantly decreased due to inhibition of the restacking of GO nanosheets and of aggregation of MWCNTs, reflecting the low propensity for crystallization of the hierarchical NF membrane. Moreover, the diffraction peak of the GO nanosheets slightly shifted from 11.02° to 10.63°, corresponding to an increase in interlayer spacing from 0.81 to 0.87 nm. Bands in the wide-scan XPS spectra of the respective membranes confirmed the increased N 1 s content in the PDDA-MWCNTs/GO membrane.

**Fig. 6:**
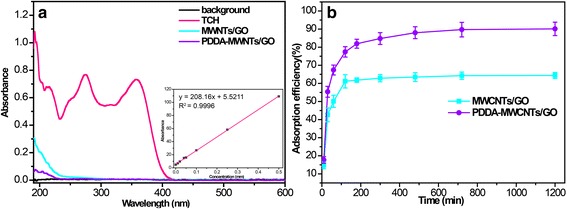
**a** UV/Vis absorption spectra of the initial TCH solution and the residue solutions obtained by filtration using MWCNTs/GO and PDDA-MWCNTs/GO membranes. The insert picture is the standard curve of concentration of TCH (10 μM, 20 μM, 40 μM, 50 μM, 100 μM, 250 μM, 500 μM). **b** The static adsorption of TCH

Figure 6a shows the UV/Vis absorption spectra of the initial TCH solution (20 mL, 500 μm) and of the filtrates obtained after passage through the MWCNTs/GO and PDDA-MWCNTs/GO membranes. After filtration through the all-carbon membranes, the solution showed lower absorption intensity in the region up to 420 nm. Combined with the inset image, the remaining concentrations of TCH after filtration through the MWCNTs/GO and PDDA-MWCNTs/GO membranes were 18.78 and 6.74 μM, respectively. The adsorption capacities could be converted into adsorption percentages, which were evaluated as 95.04% for MWCNTs/GO and 99.23% for PDDA-MWCNTs/GO, following a single filtration through each membrane. Thus, compared with the MWCNTs/GO membrane, the PDDA-MWCNTs/GO membrane caused a more pronounced decrease in absorption intensity. From these results, we can conclude that TCH filtration involves both the interfacial functional groups and a synergistic effect. In addition, the water permeation flux for the PDDA-MWCNTs/GO membrane was evaluated as 16.12 L m^− 2^ h^− 1^ bar^− 1^ after functionalization, around twice that for the MWCNTs/GO membrane. The PDDA-MWCNTs/GO membrane clearly demonstrated the best results in terms of both high adsorption capability and water permeation flux. Figure 6b shows the static adsorption of TCH. In this experiment, the mass of the PDDA-MWCNTs/GO membrane was 1 mg. After static adsorption, the amount adsorbed on the PDDA-MWCNTs/GO membrane was 436.13 mg g^−1^, confirming its high capacity for TCH removal from water.

**Fig. 7:**
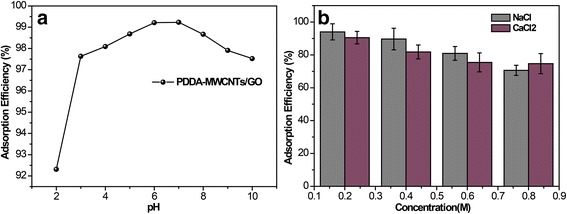
**a** Effect of adsorption of TCH at different pH. **b** Effect of adsorption of TCH in saline solution

Stability is important for practical application of NF membranes. Here, we conducted adsorption experiments in harsh environments, namely, basic, acidic [29], and ionic conditions (Fig. 7). We anticipated that pH would affect the electrostatic interaction by regulating the charges on both TCH and the membrane. It is found that the zeta potential of the membrane is around − 45 mV, while TCH has a positive and negative charge in the acid and alkaline conditions, respectively [30]. When the pH raised from 2 to 4 or from 8 to 10, the adsorption of TCH was slightly decreased (Fig. 7a). This may due to membrane swelling or electrostatic repulsion [31]. Neutral pH proved to be optimal, and all further experiments reported herein were conducted at pH 7. As we could see, for the PDDA-MWCNTs/GO membrane, the adsorption behavior was only slightly influenced. From this, it could be surmised that the main adsorption mechanism is capture of molecules in the nanochannels. As shown in Fig. 7b, the adsorption of TCH decreased with increasing salt concentration. Salt-induced membrane swelling and salting-out effects may have synergistically influenced the adsorption performance [32]. Nevertheless, the prepared membrane showed a moderate tolerance for saline ions.

We used an MB dye as a positively charged molecule to further study the adsorption mechanism. We concluded that a synergistic effect was operative, involving molecular sieving in the nanochannels, the hydrophobicity of the non-oxide nanochannels in the membrane, and charge interaction between the solute and the membrane [33]. The ζ-potential for the PDDA-MWCNTs/GO NF membrane is at around pH 7, indicating good stabilization, and it showed greater adsorption of the zwitterionic TCH (99.23%) than that of the positively charged MB dye (88.23%). We attribute this to size exclusion being the major factor in the controlled nanoscale channels [34, 35], consistent with the results of pH experiments.

## Conclusions

In summary, we have proposed a novel 3D all-carbon NF membrane with tremendous properties, namely, ultrathin nanosheets with high adsorption, stabilization with extraordinary anti-fouling properties, and rapid water permeation. The synthesis is rapid and environmentally friendly, making it a promising method for the fabrication of NF membranes. The functionalized PDDA-MWCNTs/GO NF membrane exhibited superior properties compared to the MWCNTs/GO NF membrane due to the high dispersion of MWCNTs and charge interaction between the components. The high adsorption performance can be attributed to a synergistic effect between molecular sieving, the hydrophobicity of the non-oxide nanochannels in the membrane, and the charge interaction between the solute and the membrane. The simple preparation process combined with the many extraordinary properties makes this functionalized MWCNTs/GO NF membrane a promising candidate for chemical separation applications.

## Methods/Experimental

### Materials

GO (2 mg/mL) dispersion was purchased from Nanjing XFNANO Materials Tech Co. (Nanjing, China). Pure MWCNTs with an average diameter of 20–30 nm and approximate lengths of 10–30 μm were purchased from Beijing Boyu High-tech Novel Materials Technology Co. (Beijing, China). PDDA (200,000 ≤ MW ≤ 350,000, 20 wt% in H_2_O), TCH powder (analytical standard), sodium chloride solid (NaCl, S), calcium chloride solid (CaCl_2_, S), hydrochloric acid (HCl), and anhydrous ethanol (CH_3_CH_2_OH) were purchased from Aladdin Chemical Co. (Shanghai, China). Deionized water (18 M Ω cm^−1^) used throughout the experiments was produced by a water purification system (Billerica, MA, USA).

### Apparatus

Scanning electron microscope (SEM) images of the prepared all-carbon membrane were acquired with a field-emission scanning electron microscope (FESEM, Ultra 55, Carl Zeiss, Germany). Suspensions of graphene oxide and MWCNTs were dropped onto carbon-coated copper grids, and the volatiles were evaporated under ambient conditions. Transmission electron microscopy (TEM) was performed using a Hitachi H-800 electron microscope (Japan) operated at an accelerating voltage of 200 kV. UV/Vis spectra were recorded on a Lambda-25 spectrometer (Perkin-Elmer Inc. USA). Brunauer–Emmett–Teller (BET) measurements were performed at 77 K on an Autosorb-iQ-C analyzer (Quantachrome Instruments, USA). X-ray diffraction (XRD) patterns were obtained with a Shimadzu XD-3A diffractometer (Japan), employing CuKα radiation, *λ* = 0.15418 nm. Various elements in the samples were determined by X-ray photoelectron spectroscopy (XPS, PHI 5000 Versa Probe, Japan). Static water contact angle measurements were performed at 25 °C using a contact angle meter (Rame-Hart-100, USA) employing drops of pure deionized water. Zeta potentials of the membranes were tested by means of a SurPASS Electrokinetic Analyzer (Austria) with a clamping cell at 300 mbar. A Bruker Multimode 8 atomic force microscope (AFM, Germany) was employed to characterize the prepared nanomaterials, which had been coated on a mica substrate.

### Synthesis of PDDA-Functionalized MWCNTs

PDDA-functionalized MWCNTs were prepared as described previously [36]. MWCNTs (4.0 mg) were first dispersed in deionized water (1 mL) with the aid of ultrasonication, and PDDA (10 wt%) in water was added dropwise. The centrifuged product was then washed several times with deionized water and dried in a vacuum oven at 70 °C for 24 h.

### Assembly of the MWCNT-Interposed GO (MWCNTs/GO) Membrane

MWCNTs (4.0 mg) were added to an aqueous suspension of GO (24 mL, 0.5 mg mL^−1^) under stirring and sonication. The homogeneous dispersion was then vacuum-filtered onto a porous polyvinylidene fluoride membrane with a pore size of 0.22 μm. Finally, the membrane was dried in a vacuum oven at 60 °C for 3 min and could be easily peeled from the polyvinylidene fluoride membrane after soaking with anhydrous ethanol.

### Antibiotic Adsorption Experiments Using the All-Carbon Membrane

In order to evaluate the adsorption performance towards antibiotics, TCH solution (20 mL, 500 μM) was vacuum-filtered at 0.9 bar through the prepared membrane. The concentration of the filtrate was determined by UV/Vis spectrophotometry. According to the determined concentration, then the rejection rates of the TCH molecules could be calculated through the following equation:

$$ R=\left(1-{C}_{\mathrm{p}}/{C}_{\mathrm{o}}\right)\times 100\% $$

where *C*_o_ represents the concentration of TCH in the original solution and *C*_p_ is the concentration of TCH in the permeate solution. All of the data were calculated based on the results from at least three experiments.

### Stability Experiments with the All-Carbon Membranes

TCH solids were dissolved into different pH solutions (pH = 2, 3, 4, 5, 6, 7, 8, 9, and 10) using HCl (1.0 M) or NaOH (1.0 M). The as-prepared TCH solution (20 mL, 500 μM) was filtrated through the all-carbon membrane to value its tolerance for harsh condition. To explore the all-carbon membrane’s stability in saline solution, different concentrations of NaCl and CaCl_2_ (0.1, 0.2, 0.3, 0.4, 0.5, 0.6, 0.7, 0.8, and 0.9 M) were also prepared. Then, TCH was dissolved into the above saline solution. Similarly, the TCH solutions (20 mL, 500 μM) were filtrated through the all-carbon membrane. The concentrations of all the filtrates were determined by UV/Vis spectrophotometry.

## Additional file

Additional file 1: **Figure S1.** The water contact angle of the PDDA-MWCNTs/GO membrane. Figure S2 The AFM images of (A) PDDA-MWCNTs/GO membrane and (B) GO membrane. Figure S3 (A) SEM and (B) TEM images of the PDDA-MWCNTs. (DOCX 512 kb)

## Abbreviations

AFM: Atomic force microscope
BET: Brunauer–Emmett–Teller
MWCNTs: Multi-walled carbon nanotubes
PDDA: Poly dimethyl ammonium chloride
SEM: Scanning electron microscopy
SWCNTs: Single-walled carbon nanotubes
TCH: Tetracycline hydrochloride
TEM: Transmission electron microscopy
XPS: X-ray photoelectron spectroscopy
XRD: X-ray diffraction

## References

[1] Baquero F, Martínez J-L, Cantón R (2008). Antibiotics and antibiotic resistance in water environments. Curr Opin Biotechnol.

[2] Cabello FC (2006). Heavy use of prophylactic antibiotics in aquaculture: a growing problem for human and animal health and for the environment. Environ Microbiol.

[3] Zhou KF, Xie XD, Chang CT (2017). Photocatalytic degradation of tetracycline by Ti-MCM-41 prepared at room temperature and biotoxicity of degradation products. Appl Surf Sci.

[4] Song QQ (2017). The performance of porous hexagonal BN in high adsorption capacity towards antibiotics pollutants from aqueous solution. Chem Eng J.

[5] Shannon MA, Bohn PW, Elimelech M, Georgiadis JG, Marinas BJ, Mayes AM (2008). Science and technology for water purification in the coming decades. Nature.

[6] Košutić K, Dolar D, Ašperger D, Kunst B (2007). Removal of antibiotics from a model wastewater by RO/NF membranes. Sep Purif Technol.

[7] Li SQ, Zhang XD, Huang YM (2017). Zeolitic imidazolate framework-8 derived nanoporous carbon as an effective and recyclable adsorbent for removal of ciprofloxacin antibiotics from water. J Hazard Mater.

[8] Shi S, Fan YW, Huang YM (2013). Facile low temperature hydrothermal synthesis of magnetic mesoporous carbon nanocomposite for adsorption removal of ciprofloxacin antibiotics. Ind Eng Chem Res.

[9] Mi B (2014). Materials science. Graphene oxide membranes for ionic and molecular sieving. Science (New York, NY).

[10] Zhang N, Qiu H, Si Y, Wang W, Gao J (2011). Fabrication of highly porous biodegradable monoliths strengthened by graphene oxide and their adsorption of metal ions. Carbon.

[11] Chang ZY, Deng JK, Chandrakumara GG, Yan WY, Liu JZ (2016). Two-dimensional shape memory graphene oxide. Nat Commun.

[12] Leng XH, Li WN, Luo D, Wang F (2017). Differential structure with graphene oxide for both humidity and temperature sensing. IEEE Sensors J.

[13] Lee S-H (2013). Graphene–nanotube–iron hierarchical nanostructure as lithium ion battery anode. ACS Nano.

[14] Marcano DC (2010). Improved synthesis of graphene oxide. ACS Nano.

[15] Putz KW, Compton OC, Palmeri MJ, Nguyen ST, Brinson LC (2010). High-nanofiller-content graphene oxide-polymer nanocomposites via vacuum-assisted self-assembly. Adv Funct Mater.

[16] Xu H, Ding MM, Liu S, Li Y, Shen Z, Wang K (2017). Preparation and characterization of novel polysulphone hybrid ultrafiltration membranes blended with N-doped GO/TiO_2_ nanocomposites. Polymer.

[17] Kabiri S, Tran DNH, Altalhi T, Losic D (2014). Outstanding adsorption performance of graphene-carbon nanotube aerogels for continuous oil removal. Carbon.

[18] Goh K (2015). All-carbon nanoarchitectures as high-performance separation membranes with superior stability. Adv Funct Mater.

[19] Tristán-López F (2013). Large area films of alternating graphene–carbon nanotube layers processed in water. ACS Nano.

[20] Ge JJ (2005). Multiwalled carbon nanotubes with chemically grafted polyetherimides. J Am Chem Soc.

[21] Deng JJ, You Y, Bustamante H, Sahajwalla V, Joshi RK (2017). Mechanism of water transport in graphene oxide laminates. Chem Sci.

[22] Zhang YQ, Yang XB, Wang ZX, Long J, Shao L (2017). Designing multifunctional 3D magnetic foam for effective insoluble oil separation and rapid selective dye removal for use in wastewater remediation. J Mater Chem A.

[23] Wang Z, Yang X, Cheng Z, Liu Y, Shao L, Jiang L (2017). Simply realizing “water diode” Janus membranes for multifunctional smart applications. Mater Horiz.

[24] Zhi C, Xu Y, Bando Y, Golberg D (2011). Highly thermo-conductive fluid with boron nitride nanofillers. ACS Nano.

[25] Liu Y (2017). Antifouling, high-flux oil/water separation carbon nanotube membranes by polymer-mediated surface charging and hydrophilization. J Membr Sci.

[26] Velasco LF, Guillet-Nicolas R, Dobos G, Thommes M, Lodewyckx P (2016). Towards a better understanding of water adsorption hysteresis in activated carbons by scanning isotherms. Carbon.

[27] Fan Z, Wang K, Wei T, Yan J, Song L, Shao B (2010). An environmentally friendly and efficient route for the reduction of graphene oxide by aluminum powder. Carbon.

[28] Kumar R, Ansari MO, Barakat MA (2014). Adsorption of brilliant green by surfactant doped polyaniline/MWCNTs composite: evaluation of the kinetic, thermodynamic, and isotherm. Ind Eng Chem Res.

[29] Huang L (2016). Reduced graphene oxide membranes for ultrafast organic solvent nanofiltration. Adv Mater.

[30] Li B, Zhang T (2013). Removal mechanisms and kinetics of trace tetracycline by two types of activated sludge treating freshwater sewage and saline sewage. Environ Sci Pollut Res.

[31] Dalwani M, Benes NE, Bargeman G, Stamatialis D, Wessling M (2011). Effect of pH on the performance of polyamide/polyacrylonitrile based thin film composite membranes. J Membr Sci.

[32] Luo J, Wan Y (2011). Effect of highly concentrated salt on retention of organic solutes by nanofiltration polymeric membranes. J Membr Sci.

[33] Jabbari V, Veleta JM, Zarei-Chaleshtori M, Gardea-Torresdey J, Villagran D (2016). Green synthesis of magnetic MOF@GO and MOF@CNT hybrid nanocomposites with high adsorption capacity towards organic pollutants. Chem Eng J.

[34] Liang B (2016). Membranes with selective laminar nanochannels of modified reduced graphene oxide for water purification. Carbon.

[35] Ma X-H, Yang Z, Yao Z-K, Xu Z-L, Tang CY (2017). A facile preparation of novel positively charged MOF/chitosan nanofiltration membranes. J Membr Sci.

[36] Wang S, Yu D, Dai L (2011). Polyelectrolyte functionalized carbon nanotubes as efficient metal-free electrocatalysts for oxygen reduction. J Am Chem Soc.

