# Supplementary material for: Removal of Antibiotics From Water with an All-Carbon 3D Nanofiltration Membrane
Source: Nanoscale Res Lett. 2018 May 10;13:146. doi: 10.1186/s11671-018-2555-9 (PMC5945562; doi:10.1186/s11671-018-2555-9)
Supplement: Supplementary file 1 — Figure S1. The water contact angle of the PDDA-MWCNTs/GO membrane. Figure S2 The AFM images of (A) PDDA-MWCNTs/GO membrane and (B) GO membrane. Figure S3 (A) SEM and (B) TEM images of the PDDA-MWCNTs. (DOCX 512 kb) [file 11671_2018_2555_MOESM1_ESM.docx]

**Additional file 1**

Removal of Antibiotics from Water with an All-Carbon 3D Nanofiltration Membrane

Guo-hai Yang ^1#*^, Dan-dan Bao ^1#^, Da-qing Zhang ^1^, Cheng Wang ^1^, Lu-lu Qu ^1^, Hai-tao Li ^1*^

^1^School of Chemistry and Material Science, Jiangsu Normal University, Xuzhou 221116, China

*To whom correspondence should be addressed:

E-mail: yangguohai@jsnu.edu.cn; haitao@jsnu.edu.cn

^#^ These authors contributed equally to this work.

**
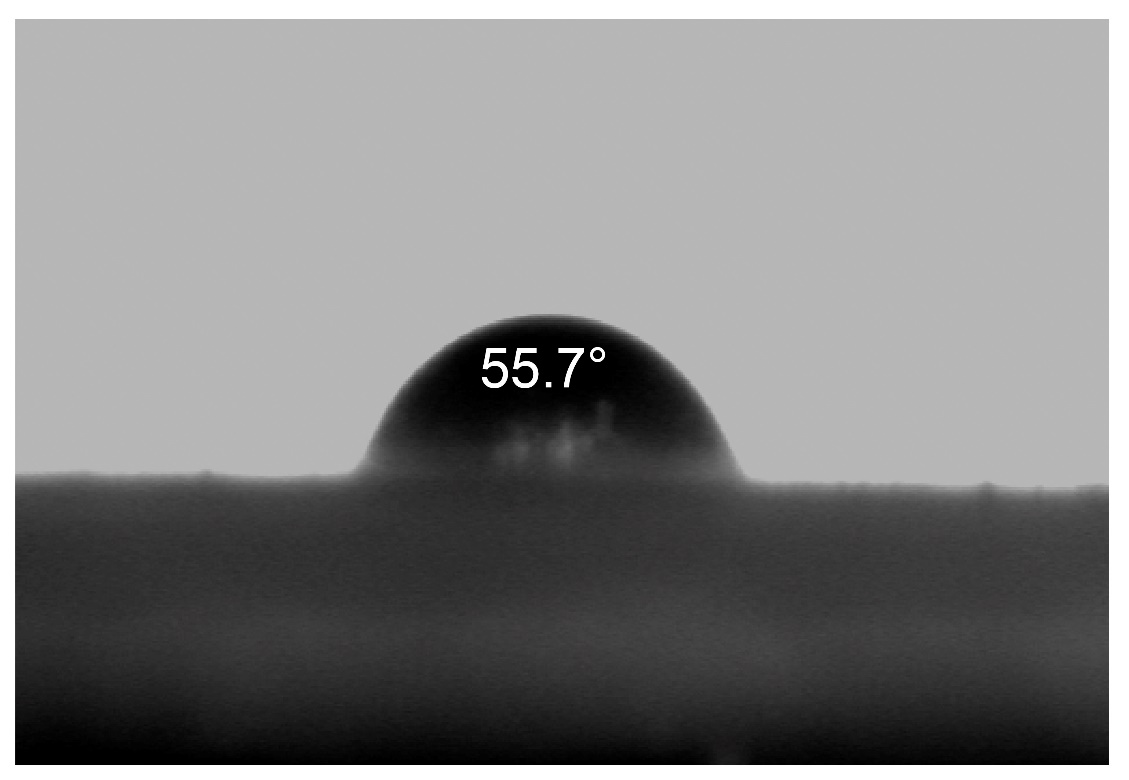
**

Figure S1 The water contact angle of the PDDA-MWCNTs/GO membrane.

As shown in figure Figure S1, the water contact angle of the top surface of as-prepared membrane is approximately 55.7°, demonstrating a hydrophilic property.


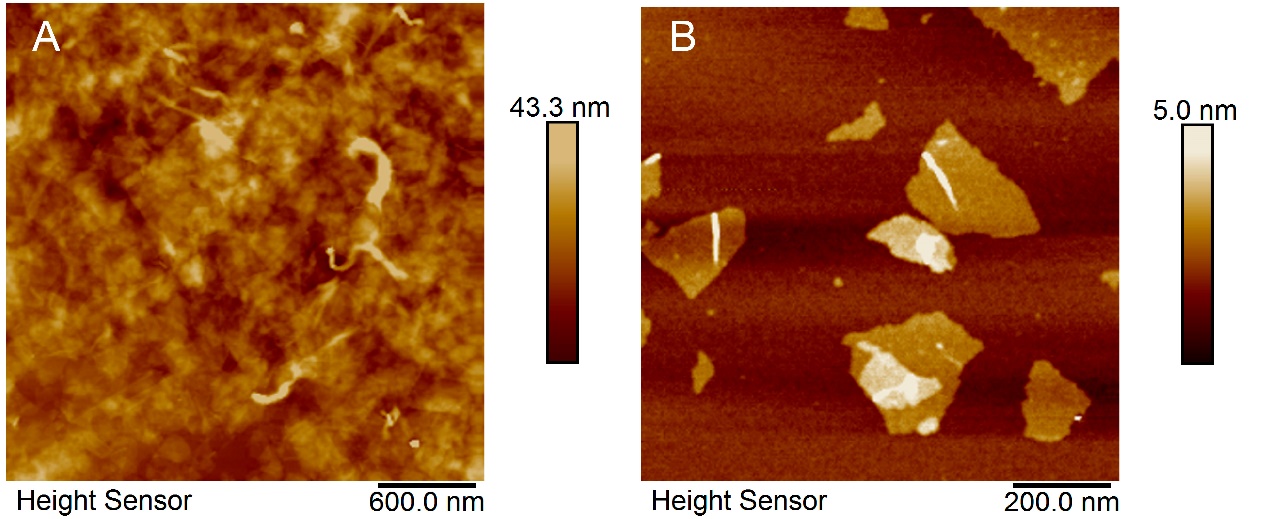


Figure S2 The AFM images of (A) PDDA-MWCNTs/GO membrane and (B) GO membrane.

In Figure S2A, AFM images suggested that the prepared membrane was much rough than GO membranes (Figure S2B). Because of the inserted CNTs, many wrinkles could be produced compared to the GO membrane. **
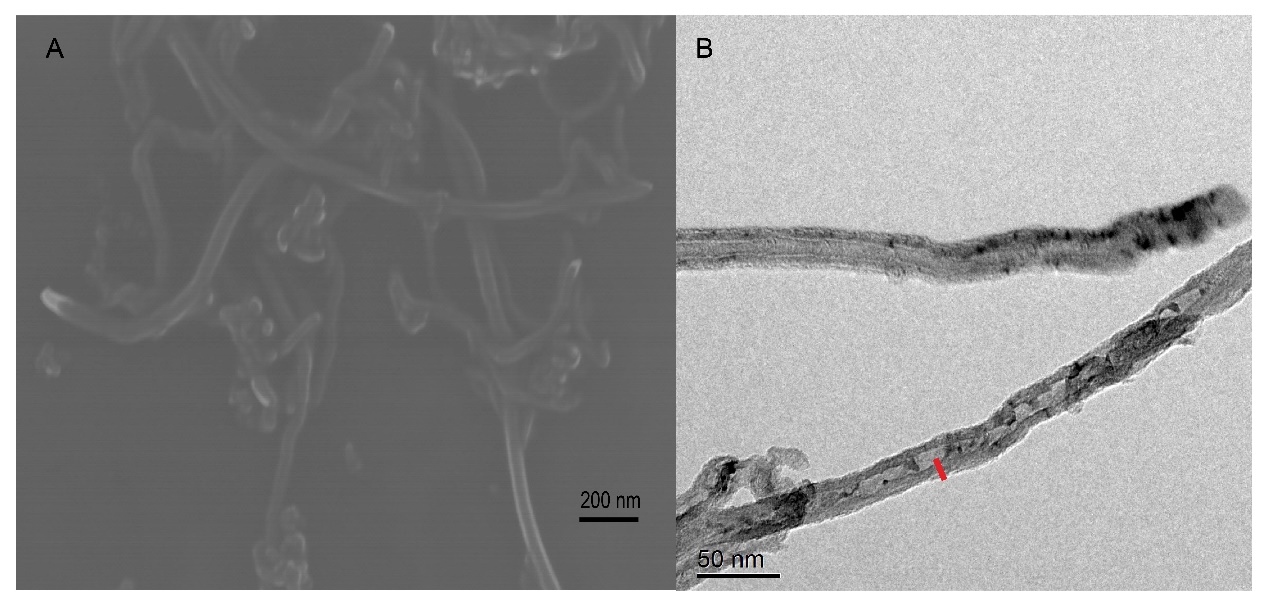
**

Figure S3 (A) SEM and (B) TEM images of the PDDA-MWCNTs.
